# Genetic by environmental variation but no local adaptation in oysters (*Crassostrea virginica*)

**DOI:** 10.1002/ece3.2614

**Published:** 2016-12-22

**Authors:** A. Randall Hughes, Torrance C. Hanley, James E. Byers, Jonathan H. Grabowski, Jennafer C. Malek, Michael F. Piehler, David L. Kimbro

**Affiliations:** ^1^ Marine Science CenterNortheastern UniversityNahantMAUSA; ^2^ Odum School of EcologyUniversity of GeorgiaAthensGAUSA; ^3^ Institute of Marine SciencesUniversity of North Carolina at Chapel HillMorehead CityNCUSA

**Keywords:** adaptive genetic variation, countergradient variation, diversity, intraspecific variation, local adaptation, oyster

## Abstract

Functional trait variation within and across populations can strongly influence population, community, and ecosystem processes, but the relative contributions of genetic vs. environmental factors to this variation are often not clear, potentially complicating conservation and restoration efforts. For example, local adaptation, a particular type of genetic by environmental (G*E) interaction in which the fitness of a population in its own habitat is greater than in other habitats, is often invoked in management practices, even in the absence of supporting evidence. Despite increasing attention to the potential for G*E interactions, few studies have tested multiple populations and environments simultaneously, limiting our understanding of the spatial consistency in patterns of adaptive genetic variation. In addition, few studies explicitly differentiate adaptation in response to predation from other biological and environmental factors. We conducted a reciprocal transplant experiment of first‐generation eastern oyster (*Crassostrea virginica*) juveniles from six populations across three field sites spanning 1000 km in the southeastern Atlantic Bight in both the presence and absence of predation to test for G*E variation in this economically valuable and ecologically important species. We documented significant G*E variation in survival and growth, yet there was no evidence for local adaptation. Condition varied across oyster cohorts: Offspring of northern populations had better condition than offspring from the center of our region. Oyster populations in the southeastern Atlantic Bight differ in juvenile survival, growth, and condition, yet offspring from local broodstock do not have higher survival or growth than those from farther away. In the absence of population‐specific performance information, oyster restoration and aquaculture may benefit from incorporating multiple populations into their practices.

## 1 Introduction

Substantial trait divergence can occur across populations at small spatial scales, even in the absence of significant population genetic structure (Conover, Clarke, Munch, & Wagner, 2006; Richardson, Urban, Bolnick, & Skelly, 2014; Sanford & Kelly, 2011). This research highlights the need to better understand the distribution of intraspecific functional variation within and across populations and whether it results from genetic variation (G), environmental differences (E), or a combination of the two (G*E). Although G*E interactions can take many forms, one of the most common expectations is that of local adaptation, in which local genotypes will have higher fitness in their native habitat than foreign populations from farther away (Anderson, Perera, Chowdhury, & Mitchell‐Olds, 2015; Kawecki & Ebert, 2004; Richardson et al., 2014; Sanford & Kelly, 2011). Local adaptation measures the degree that adaptive genetic variation matches environmental variation and thus necessarily requires testing multiple populations across multiple environments (Blanquart, Kaltz, Nuismer, & Gandon, 2013; Kawecki & Ebert, 2004). There is a large body of evidence demonstrating the importance of local adaptation in terrestrial and freshwater systems (De Meester, 1996; Linhart & Grant, 1996; Rua et al., 2016), but we know comparably less about adaptive genetic variation in marine species (Sanford & Kelly, 2011; Sotka, 2005), many of which have greater mean propagule dispersal distance than their terrestrial counterparts (Kinlan & Gaines, 2003). Determining whether marine species that are economically and ecologically important are locally adapted is also critically important to ongoing conservation, restoration, and management efforts.

Another specific form of G*E variation is countergradient variation, in which genetic variation counteracts environmental influences to decrease phenotypic variation across an environmental gradient (Conover & Schultz, 1995). For instance, in a common environment, aquatic, terrestrial, and marine populations from cooler environments and/or higher latitudes often have higher rates of growth, respiration, or development than populations from lower latitudes (Conover & Schultz, 1995; Conover and Present 1990; Laugen, Laurila, Rasanen, & Merila, 2003; Sanford & Kelly, 2011; Sears & Angilletta, 2003). Countergradient variation in response to temperature is common in species with broad geographic ranges (Conover et al., 2006; Sanford & Kelly, 2011). Species also demonstrate more complex geographic patterns of adaptation in response to spatially varying selection (Sanford & Kelly, 2011; Thompson, 1999). For example, predation intensity can vary across a range of spatial scales from within sites (e.g., along an elevation gradient; Connell, 1961) to across sites (e.g., across wave exposed and protected sites; Menge, 1976) to across regions (e.g., from low to high latitudes; Stachowicz & Hay, 2000; Sanford & Kelly, 2011). Similar persistent variation can occur in abiotic selective factors, such as nutrients, pH, and hypoxia, suggesting that adaptation at a range of scales is possible and that identifying the specific selective factors may be challenging (Sanford & Kelly, 2011). Explicitly testing fitness in the presence and absence of predation across multiple environments can provide valuable information regarding the abiotic and biotic factors contributing to G*E variation.

Reciprocal transplant experiments, in which different populations are transplanted to multiple common experimental environments in the field, are the most effective method for testing for G*E variation (Blanquart et al., 2013; Conover & Schultz, 1995; Kawecki & Ebert, 2004; Sanford & Kelly, 2011). The strength of reciprocal transplant experiments is that they test whether patterns of adaptive genetic variation are consistent across multiple populations and environments, and they incorporate natural environmental variation (Blanquart et al., 2013; Sanford & Kelly, 2011). Similar to provenance trials in forestry research, reciprocal transplant experiments enhance the ability to detect G*E interactions (Anderson et al., 2015). Despite their strengths, field reciprocal transplant experiments are relatively rare, particularly across more than two sites, due in part to their logistical complexity and/or geographic restrictions on relocating organisms (Blanquart et al., 2013; Kawecki & Ebert, 2004; Sanford & Kelly, 2011). This gap is most noticeable in marine ecosystems (but see Blanchette, Miner, & Gaines, 2002; Burford, Scarpa, Cook, & Hare, 2014; Bible & Sanford, 2016), where it was traditionally assumed that local adaptation was rare due to perceived high dispersal among populations (Grosberg & Cunningham, 2001; Marshall, Monro, Bode, Keough, & Swearer, 2010; Sanford & Kelly, 2011; Warner, 1997). When they are utilized, reciprocal transplant experiments often rely on field‐collected individuals, complicating their interpretation as preconditioned environmental effects affecting early life stages may persist in these individuals long after they are transplanted to a new site (Sanford & Kelly, 2011). In contrast, tests using offspring from multiple populations reared in a common environment and then transplanted to the field provide a much stronger test of local adaptation (Bible & Sanford, 2016; Sanford & Kelly, 2011).

**Figure 1:**
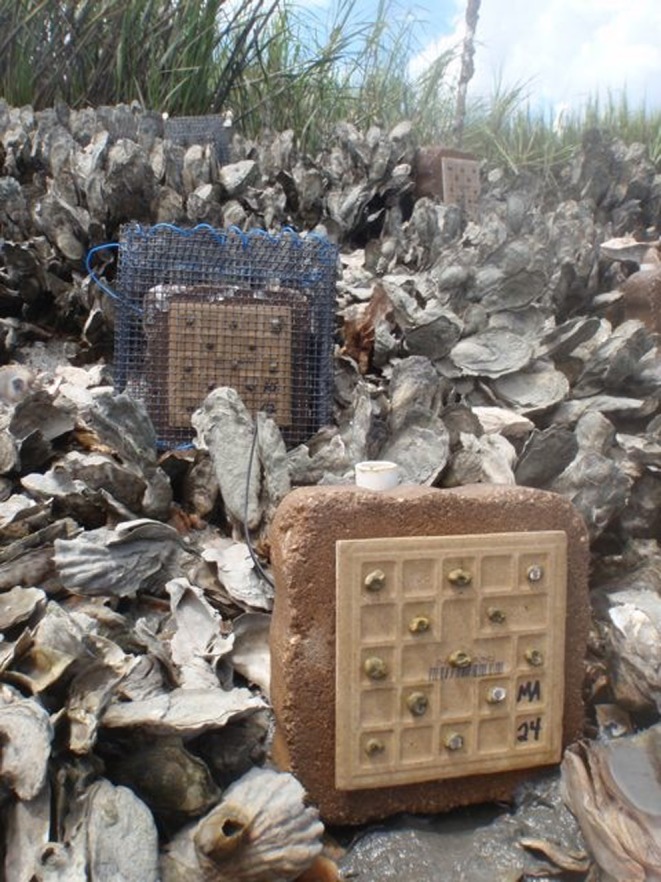
*Crassostrea virginica* reciprocal transplant experiment. Juvenile oysters were affixed to experimental tiles in no cage (front tile), cage (back tile), or cage control (not pictured) treatments and deployed on natural oyster reefs in NC, GA (pictured), and FL. After 6 weeks, we measured oyster survival, growth, condition, and parasite prevalence

Eastern oysters (*Crassostrea virginica*) provide a well‐studied, experimentally tractable, and ecologically important species with which to test for the presence of adaptive genetic variation and countergradient selection in marine ecosystems (Figure 1). Despite a 2–3 week planktonic larval stage (Kennedy, 1996), oysters exhibit significant genetic differentiation among populations in multiple regions of their distribution (e.g., north and south of Cape Canaveral, FL: Reeb & Avise, 1990; Hare & Avise, 1996; Chesapeake Bay: Rose, Paynter, & Hare, 2006). In addition, Burford et al. (2014) demonstrated local adaptation in oyster populations on either side of the genetic break in Cape Canaveral, FL, with stronger local adaptation in the north. Although a prior study found that oysters in the southeastern USA exhibited little population structure (Diaz‐Ferguson, Robinson, Silliman, & Wares, 2010), this apparent homogeneity across neutral genetic markers does not preclude adaptive genetic variation in fitness (Conover et al., 2006; Marshall et al., 2010; Sanford & Kelly, 2011). In fact, our prior work in the SAB demonstrated significant environmental and ecological variation in oyster reef communities across the sites included in this study (Byers et al., 2015; Kimbro, Byers, Grabowski, Hughes, & Piehler, 2014). For instance, temperature increases with decreasing latitude from North Carolina (NC) to Florida (FL) in all seasons except summer (Byers et al., 2015), suggesting the potential for countergradient selection in response to the abiotic environment. In addition, predator diversity and predation pressure vary across our sites in the SAB (Gehman et al., 2016), creating the potential for selection in response to the biotic environment as well. Finally, we found effects of the identity and number of juvenile oyster populations on recruitment, growth, and survival at the local scale (<1 m^2^: Hanley, Hughes, Williams, Garland, & Kimbro, 2016; see also Smee, Overath, Johnson, & Sanchez, 2013), highlighting the potential for G*E in our study area.

**Figure 2:**
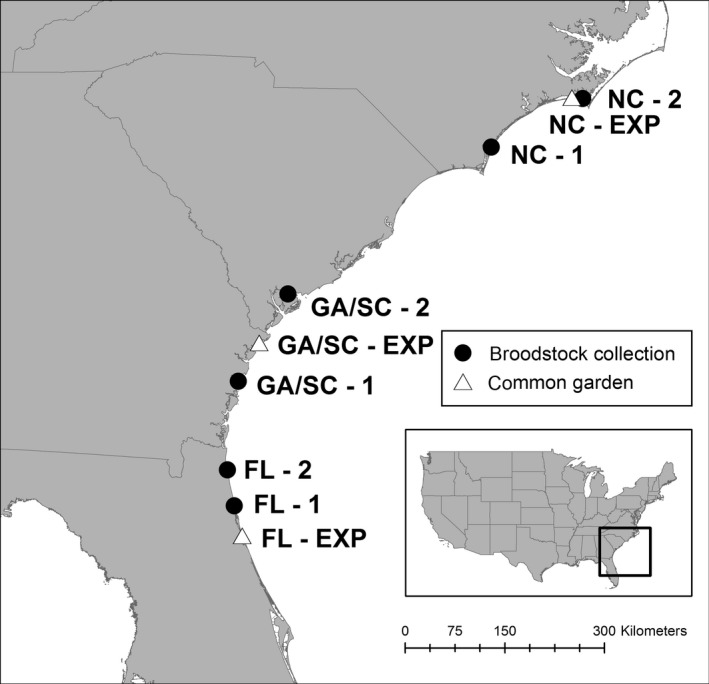
Map of study region in the southeastern Atlantic Bight (SAB). Six sites where adult oysters were collected for broodstock are indicated in closed circles: St. Augustine, FL (FL‐1), Jacksonville, FL (FL‐2), Sapelo Island, GA (GA/SC‐1), Ace Basin, SC (GA/SC‐2), Masonboro Inlet, NC (NC‐1), and Bogue Sound, NC (NC‐2). The three experimental sites are indicated in open triangles

To distinguish between genetic and environmental variation in oyster fitness across populations, we conducted a reciprocal transplant experiment at three sites using juvenile oysters (i.e., spat) produced in a single hatchery by adult broodstock from six collection sites spanning 1000 km in the SAB (Figure 2). As with plants (Anderson et al., 2015) and other invertebrates (Gosselin & Qian, 1997; Levinton, 2014), oysters experience high mortality at the juvenile stage due to both biotic and abiotic forces postsettlement, making this early life history stage critical for understanding local adaptation (Anderson, 2016). Thus, we conducted our experiment in the summer during this period of high mortality, and we included both caged and uncaged oysters to distinguish the effects of predation from other sources of mortality. We measured multiple fitness responses of juvenile oysters at the end of our six‐week field experiment, including survival in the presence and absence of predation; growth and condition in the absence of predation; and parasite prevalence in the absence of predation. Specifically, we addressed the following questions: (i) Is there genetic and/or environmental variation in juvenile oyster performance in the SAB? (ii) Is there evidence for local adaptation? and (iii) Do oysters exhibit countergradient selection across the temperature gradient from NC to FL? Understanding the presence and scale of adaptive variation is particularly important for ecologically important and economically valuable species such as oysters that are harvested in the wild, grown in aquaculture, and are the focus of extensive conservation and restoration efforts.

## 2 Materials and Methods

### 2.1 Study system

Eastern oysters (*Crassostrea virginica*) are common intertidally throughout the southeastern USA (Dame, 1972). Because they settle gregariously, oysters create structured habitat in an otherwise soft‐sediment environment, and these oyster reefs serve as a key habitat for a range of recreationally and commercially important estuarine fishes and invertebrates (Bahr & Lanier, 1981; Beck et al., 2011; Lenihan & Peterson, 1998; Peterson, Grabowski, & Powers, 2003; Wells, 1961). In addition to harboring a diverse community of animals, oysters filter large volumes of water, removing excess nitrogen and filtering down the abundance of harmful algae and microbes (Dame, 1972; Grabowski et al., 2012; Newell, 1988; Piehler & Smyth, 2011). Eastern oysters are subject to infection by multiple parasites, which can have substantial impacts on oyster abundance. *Perkinsus marinus*, the causative agent of Dermo disease, is a prevalent pathogen of *C. virginica* along the Atlantic and Gulf coasts of the USA and is commonly associated with high mortality (Burreson & Ragone‐Calvo, 1996; Ray, 1996). *Haplosporidium nelsoni*, the causative agent of multinucleated sphere unknown (MSX) disease, has been associated with epizootic outbreaks along much of the Atlantic Coast (Ford & Haskin, 1982; Sunila, Karolus, & Volk, 1999). Patterns of parasite prevalence and infection intensity in oysters depend on environmental conditions, including temperature and salinity (Breitburg et al., 2015; Burreson & Ragone‐Calvo, 1996; Cook, Folli, Klinck, Ford, & Miller, 1998). Furthermore, climate change is contributing to epizootic outbreaks and parasite range expansion (Harvell et al., 1999, Ford & Chintala, 2006), making it important to consider the role of local adaptation in parasite resistance of the eastern oyster.

### 2.2 Establishing oyster families

In April 2012, we collected 100 adult oysters (≥75‐mm shell length) from 3–5 separate reefs at each of six sites in the SAB (Figure 2): St. Augustine, Florida (FL‐1), Jacksonville, Florida (FL‐2), Sapelo Island, Georgia (GA/SC‐1), Ace Basin, South Carolina (GA/SC‐2), Masonboro, North Carolina (NC‐1), and Middle Marsh, North Carolina (NC‐2). These sites were part of a broader study examining geographic variation in oyster reef community structure and function (Kimbro et al., 2014; Byers et al., 2015), and all are north of the genetic break that occurs in Cape Canaveral, FL (Hare & Avise, 1996; Burford et al., 2014). The adult oysters were held in flowing seawater tanks or suspended in cages from docks in their home region for 2‐3 weeks until a subset of 30 oysters from each site could be tested and certified as being free of parasite infection using microscopy at the Aquatic Animal Health Laboratory at Florida Atlantic University. The remaining 70 oysters were then shipped on ice to a single hatchery facility in Florida (Research Aquaculture Inc., Tequesta, FL; >300 km south of our southernmost FL site) at the end of April and used as broodstock to produce six separate oyster cohorts (one cohort per adult oyster collection site). From their arrival at the hatchery, the adult oysters and their offspring were held in separate flow‐through seawater systems under identical conditions to prevent cross‐contamination among cohorts and avoid the confounding effects of environmental variability. The adults were held for two weeks until they were ready to spawn. All cohorts were then manually spawned on the same day.

Following spawning, the larvae from each cohort were held in separate seawater systems with identical food concentration until they settled (~three weeks). They were then moved into a nursery facility at the same hatchery, again under flow‐through seawater conditions (salinity = 32 ppt, temperature = 30°C) within the range experienced in the field at all sites (Byers et al., 2015). Some cohorts produced more juvenile oysters than others, despite following the same procedure for all. To maintain consistency in their growing conditions in the hatchery, in mid‐June, we collected a random sample of each cohort and discarded the rest to yield similar total abundances across cohorts.

### 2.3 Field experiment

At each of our three experimental sites (described below; see also Figures 1, 2), we deployed 18 tiles (three tiles of each of the six cohorts) to each of nine natural intertidal oyster reefs separated by 100–200 m (total = 162 tiles per site). Low spat production in the FL‐1 cohort during the hatchery/spawning process limited replication of this cohort to four reefs per experimental site (12 tiles total). Tiles were attached to concrete pavers (12*12 cm) using aquarium‐safe silicone. On each reef, the three tiles from each cohort were haphazardly assigned to one of the three predation treatments: full cage (mesh size = 12 × 12 mm openings), partial cage (consisting of two mesh sides and a partial top, to control for caging artifacts), or no cage. We then divided each reef into a 2 m × 3 m grid and assigned each tile to one of 18 locations within the grid in a completely randomized design. Each tile was centered within its grid position and separated by at least 0.25 m from neighboring tiles on the reef. To reduce negative effects of sedimentation, we positioned the tiles vertically by securing the back of the concrete paver to rebar stakes.

We chose one experimental site within each subregion of the SAB (Figure 2): Pine Knoll Shores, NC (NC); Skidaway Institute, GA (GA); Marineland, FL (FL). Experimental sites were separated from the two broodstock collection sites in the same subregion by an average of 60 km (range = 10–110 km) and are considered the home site for those collection sites. Because oysters have planktonic larvae and past analyses indicate little fine‐scale genetic structure in the SAB region (Diaz‐Ferguson et al., 2010), we chose to test local adaptation at these broader spatial scales. Experimental tiles were distributed across nine reefs (treated as a statistical block) within each site to control for local variation in biotic and abiotic conditions. For more information on oyster reefs at these sites, see Kimbro et al. (2014) and Byers et al. (2015).

At the end of June (June 25), the six cohorts were transferred to a common flow‐through facility at the Whitney Marine Biological Laboratory (MBL) in St. Augustine, FL. On July 9, the spat from each cohort were divided into three equal portions; two of these portions were express shipped to laboratory facilities (Skidaway Institute of Oceanography, UNC Institute of Marine Sciences) near our GA and NC experimental sites, respectively (Figure 2); the remaining portion was packaged similarly for 24 hr and maintained at the Whitney MBL to control for any adverse effects of shipping. Beginning on July 11, personnel in each subregion affixed spat to ceramic tiles (10*10 cm) using the marine adhesive Z‐spar; each tile had 12 spat all from a single cohort. Tiles were held in flow‐through seawater tables for less than 48 hours until they could be deployed to the field. Prior to deployment, we measured shell length of each spat.

Six weeks after deployment, we retrieved all tiles from the field and returned them to flow‐through tables at the laboratory facility in that region. Each live oyster was counted and measured. We used the number of live oysters at the end of our experiment as our metric of survival: Survival on partial cage and uncaged tiles represented the response to both predation and other sources of mortality (e.g., parasite infection, abiotic stress), whereas survival on caged tiles represented the response to all sources of mortality except predation. We calculated growth as the average change in size of individual oysters per caged tile (in the absence of predation) over the course of the experiment. Tiles were removed from the concrete pavers and frozen for later analyses of parasite infection and condition.

### 2.4 Laboratory analyses

We divided the number of living oysters from the cage treatments in half for analyses of condition index (CI) and parasite prevalence. For consistency, all CI analyses were conducted at the Florida State University Coastal and Marine Laboratory. Oysters were carefully removed from each tile, cleaned of mud, oyster recruits, and other epifauna, and weighed to get total mass. After removal, the length and width of each top valve were measured. The tissue was then removed from the shells and weighed to obtain tissue wet mass and dry mass (dried for 24 hr at 70 C). Because our experimental oysters were affixed to tiles, we could not get reliable estimates of bottom valve mass typically used in estimates of CI; thus, we calculated CI as oyster tissue dry mass in grams per top valve area (length*width) in cm. Results were consistent if cubed top valve length was used as the denominator in place of top valve area.

All parasite analyses at the end of our experiment were conducted at the University of Georgia. Gill and mantle tissue were sterilely collected from each oyster and frozen until DNA extraction with Qiagen DNeasy Blood & Tissue Kits (Qiagen, Valencia, CA, USA) according to the manufacturer's protocol. Briefly, samples were lysed for 10 min at 70°C, washed, precipitated, and lastly eluted using AE buffer (Qiagen) to produce a minimum of 200 μl of oyster DNA. Extracted DNA was stored at −20°C. The presence of *Perkinsus marinus* (Dermo) and *Haplosporidium nelsoni* (MSX) was determined using conventional polymerase chain reaction (PCR), which affords greater sensitivity and parasite detection than traditional histological methods (Ford, Allam, & Xu, 2009). The primer set for *P. marinus* detection was adapted from De Faveri, Smolowitz, and Roberts (2009), targeting the ITS region (F: 5′–CGCCTGTGAGTATCTCTCGA‐3′, R: 5′–GTTGAAGAGAAGAATCGCGTGAT‐3′). The primer set for *H. nelsoni* detection was adapted from Stokes, Siddall, and Burreson (1995) (MSX B: 5′‐ATGTGTTGGTGACGCTAACCG‐3′) and Renault et al. (2000) (MSX A: 5′‐CGACTTTGGCATTAGGTTTCAGAC C‐3′). PCR mixtures of 25 μl consisted of 12.5 μl GoTaq 2X Green Master Mix (Promega), 8 μl nuclease free water (Promega), 1.5 μl of 10 mg/ml bovine serum albumin (New England Biolabs), 0.5 μl each of 10 μM forward and reverse primer, and 2 μl template DNA. Amplification was performed in an Eppendorf Mastercycler with the following programs: 35 cycles of 94°C for 1 min, 59°C for 1 min, and 72°C for 3 min with an initial denaturation step at 94°C for 5 min and a final extension step at 72°C for 5 min for *P. marinus* and 30 cycles of 94°C for 30 s, 59°C for 30 s, and 72°C for 1.5 min, with an initial denaturation step at 94°C for 4 min and a final extension step at 72°C for 5 min for *H. nelsoni*. Positive controls as determined by microscopy for *P. marinus* and *H. nelsoni* were obtained from J. Malek and N. Stokes, respectively. Amplified products were run through a 1.5% agarose gel containing GelRed nucleic acid stain (Biotium) and viewed with a UV transilluminator. All unknown samples and positive and negative controls were run in duplicate.

### 2.5 Statistical analyses

The metrics of fitness tested in our analyses included the following: survival in the presence of all sources of mortality (survival in no cage and partial cages at the end of the experiment, with average initial size as a covariate, modeled with a binomial generalized linear model (GLM) with logit link), survival due to sources of mortality other than predation (survival in cages at the end of the experiment, with average initial size as a covariate, modeled with a binomial GLM with logit link), and growth in the absence of predation (average final size of individual oysters per cage tile, with the average initial size per cage tile and number of surviving oysters per cage tile as covariates). We also examined oyster condition (oyster tissue dry mass in grams / top valve area in cm) and parasite prevalence (proportion of infected individuals per cohort per site) to help interpret the growth and survival results.

We used a model selection approach to examine whether oyster responses varied across predation treatments and/or experimental sites (i.e., G × E) using a series of models testing the individual, additive, and interactive fixed effects of experimental site and oyster cohort. We used the difference between the sample size corrected Akaike information criterion (AICc) of a particular model and the lowest AICc observed (the AIC difference, or ∆AIC) to determine which of the candidate models best explained the observed data (Bolker, 2008; Burnham & Anderson, 2002). We also calculated the Akaike weight as the model likelihood normalized by the sum of all model likelihoods, with values close to 1.0 indicating greater confidence in the model. We included covariates of initial size (for survival and growth) and number of surviving oysters (for growth), and a random effect of experimental reef nested in site.

Because the best model for survival and growth included a significant site*cohort interaction, we also examined three components of local adaptation: (i) home vs. away (HA), (ii) local vs. foreign (LF), and (iii) sympatric vs. allopatric (SA; Blanquart et al., 2013). HA compares the survival or growth of a cohort at its home experimental site to the average mean survival or growth of the cohort when transplanted to the other two experimental sites. LF, in contrast, compares the survival or growth of a focal cohort at its home experimental site to the average survival or growth of all foreign cohorts at that experimental site. Thus, positive values of HA and LF indicate local adaptation. HA and LF were calculated for each cohort using the equations in Blanquart et al. (2013), and then averaged across cohorts to determine whether the average value (± 95% CI) differed from zero. Because the covariates were significant, these calculations were conducted on the residuals of the model including the covariates; residuals are presented in the survival and growth figures. The SA comparison provides a more powerful test for local adaptation by comparing the mean survival or growth of each cohort in sympatry (i.e., at home) vs. that of each cohort in allopatry (SA test; Blanquart et al., 2013). We ran linear models on the cohort means that included fixed effects of experimental site, oyster cohort, and the sympatric–allopatric contrast. A significant SA effect is indicative of adaptation (Blanquart et al., 2013).

To determine whether there was spatial structure underlying the GxE interaction that we missed when grouping cohorts by region, we also examined whether distance of the broodstock collection site to each experimental site could explain variation in survival or growth. We used Google Earth to calculate the straight‐line distance between each broodstock collection site and each experimental site. Our collection and experimental sites were proximal to the mouths of estuaries, so this distance is a potential proxy for their degree of connectedness. We then used model selection to examine whether distance from broodstock site to experimental site predicted survival or growth, using likelihood ratio tests to compare a null model with a random effect of experimental reef with separate nested models adding linear or quadratic effects of distance.

We also examined the evidence for countergradient variation in growth rate at each experimental site. Specifically, we compared nested models including linear and quadratic effects of home latitude (fixed factor) as a predictor of growth using likelihood ratio tests. We included average initial size and number of surviving oysters as covariates, with a random effect of experimental reef in the model. A positive linear relationship between home latitude and growth would be consistent with countergradient selection.

Analyses were run in R software (version 3.0.2) using the packages bbmle, glm, and lme4.

## 3 Results

### 3.1 Genetic and environmental variation in oyster fitness

**Figure 3:**
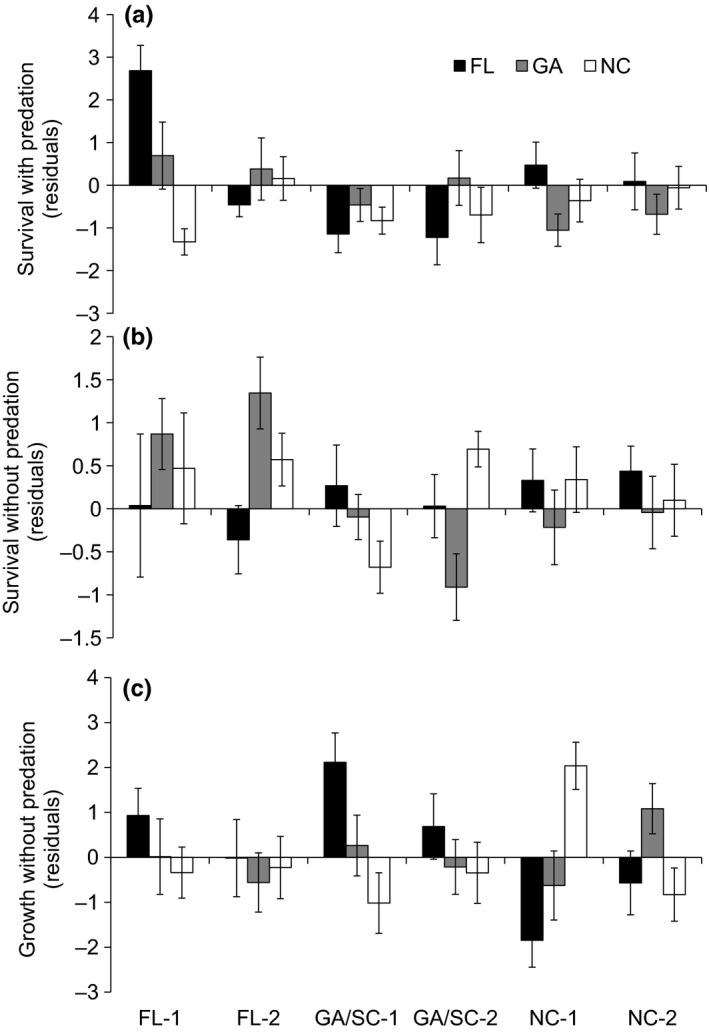
Mean (±SE) oyster survival and growth for six oyster cohorts at the FL (black bars), GA (gray bars), and NC (white bars) experimental sites. (A) Survival in partial cage treatments; (B) survival in cage treatments; (C) growth in cage treatments. The residuals of survival after accounting for initial oyster size and the residuals of growth after accounting for initial oyster size and the number of surviving oysters are presented

Oyster survival was very low in the uncaged treatments (mean[SE] percent = 4.6[1.2]), precluding formal analyses; thus, we focus only on the partial cage (with predation) and cage (without predation) treatments. Survival differed across these predation treatments and across cohorts at the three experimental sites (site*cohort*cage treatment model: Akaike weight = 1.0; Table 1). With predation (partial cages), oyster survival varied by experimental site and oyster cohort (site*cohort model: Akaike weight = 1.0; Figure 3A, Table 1). Survival was highest for the FL‐1 (mean[SE] percent = 60.4[18.7]) and NC‐1 (mean[SE] percent = 27.8[11.0]) cohorts in FL, and low for GA/SC‐1 (mean[SE] percent = 8.3[2.4]) and GA/SC‐2 (mean[SE] percent = 11.2[3.7]) at all sites (Figure 3A). Without predation (cages), survival was generally high (mean[SE] percent = 84.8[1.5]), but it differed by experimental site and oyster cohort (site*cohort model: Akaike weight = 0.44; Figure 3B, Table 1). Survival without predation was highest for FL‐2 in GA (mean[SE] percent = 87.9(3.9) and GA/SC‐2 in NC (mean[SE] percent = 88.9[2.4]), and lowest for GA/SC‐1 in NC (mean[SE] percent = 62.0[4.4]) and GA/SC‐2 in GA (mean[SE] percent = 63.5[6.1]; Figure 3B). Initial oyster size and spatial variation across reefs within sites also had substantial predictive power for survival without predation (null model: dAIC = 0.5, Akaike weight = 0.35; Table 1).

**Table 1:** Results of nested linear models for the effects of site, oyster cohort, and caging treatment on oyster vital rates

Response variable	Model	df	dAIC	Weight
Survival across predation treatments—binomial distribution	Site * Cohort * Predation treatment + Initial oyster size + (Site/Reef)	39	0.0	1.000
	Site * Cohort + Predation treatment + Initial oyster size + (Site/Reef)	22	51.3	<0.001
	Site + Cohort * Predation treatment + Initial oyster size + (Site/Reef)	17	79.3	<0.001
	Site + Cohort + Predation treatment + Initial oyster size + (Site/Reef)	12	87.8	<0.001
	Site + Predation treatment + Initial oyster size + (Site/Reef)	7	121.9	<0.001
	Cohort + Predation treatment + Initial oyster size + (Site/Reef)	10	84.6	<0.001
	Cohort + Initial oyster size + (Site/Reef)	9	1890.7	<0.001
	Site + Initial oyster size + (Site/Reef)	6	1907.1	<0.001
	Predation treatment + Initial oyster size + (Site/Reef)	5	118.5	<0.001
	Initial oyster size + (Site/Reef)	4	1904.1	<0.001
Partial cage survival—binomial distribution	Site * Cohort + **Initial oyster size** + **(Site/Reef)**	21	0.0	1.000
	Site + Cohort + Initial oyster size + (Site/Reef)	11	42.2	<0.001
	Site + Initial oyster size + (Site/Reef)	6	78.4	<0.001
	Cohort + Initial oyster size + (Site/Reef)	9	41.1	<0.001
	Initial oyster size + (Site/Reef)	4	77.9	<0.001
Cage survival—binomial distribution	Site * Cohort + **Initial oyster size** + **(Site/Reef)**	21	0.0	0.439
	Site + Cohort + Initial oyster size + (Site/Reef)	11	5.9	0.023
	Site + Initial oyster size + (Site/Reef)	6	3.2	0.088
	Cohort + Initial oyster size + (Site/Reef)	9	2.9	0.103
	**Initial oyster size** + **(Site/Reef)**	4	0.5	0.347
Growth (cage treatments)	Site * Cohort + **Initial oyster size** + **Cage survival** + **(Site/Reef)**	23	0.0	1.0
	Site + Cohort + Initial oyster size + Cage survival + (Site/Reef)	13	26.5	<0.001
	Site + Initial oyster size + Cage survival + (Site/Reef)	8	22.7	<0.001
	Cohort + Initial oyster size + Cage survival + (Site/Reef)	11	28.8	<0.001
	Initial oyster size + Cage survival + (Site/Reef)	6	25.4	<0.001
Condition (cage treatments)	Site * Cohort + (Site/Reef)	21	142.8	<0.001
	Site + Cohort + (Site/Reef)	11	22.8	<0.001
	Site + (Site/Reef)	5	71.3	<0.001
	**Cohort** + **(Site/Reef)**	9	0.0	1.000
	(Site/Reef)	4	50.4	<0.001

Bold indicates best model. Parentheses denote random effects. dAIC is the difference between the AICc of a particular model compared to the lowest AICc observed. The Akaike weight is calculated as the model likelihood normalized by the sum of all model likelihoods; values close to 1.0 indicate greater confidence in the selection of a model.

**Figure 4:**
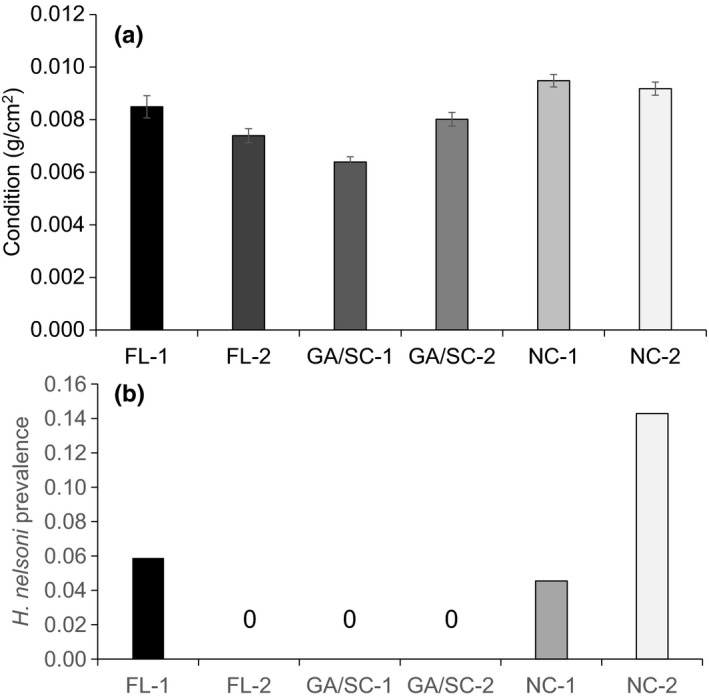
Mean (±SE) (A) oyster condition and (B) *H. nelsoni* prevalence at the end of the experiment across oyster cohorts. The lack of bars in panel (B) represents zero infection (not missing data). Prevalence was calculated at the cohort level, so there is no estimate of error

Oyster growth in the caged treatments also differed interactively by experimental site and oyster cohort (site*cohort model: Akaike weight = 1.0; Figure 3C, Table 1). The GA/SC‐1 cohort grew faster in FL (mean[SE] change in shell length = 13.36[1.29] mm) than in NC (mean[SE] change in shell length = 7.16[0.92] mm), whereas the NC‐1 cohort grew faster in NC (mean[SE] change in shell length = 10.39[0.64] mm) than in FL (mean[SE] change in shell length = 8.22[1.22] mm; Figure 3C). The NC‐2 cohort in NC (mean[SE] change in shell length = 7.22[0.86] mm) also had low growth overall (Figure 3C). Oyster condition in the absence of predation, in contrast, differed primarily by oyster cohort (cohort model: Akaike weight = 1.0; Figure 4A, Table 1). GA/SC‐1 oysters had the lowest condition (mean[SE] g/cm^2^ = 0.006[0.0003]), while NC oysters had highest condition (NC‐1: mean[SE] g/cm^2^ = 0.009[0.0002]; NC‐2: mean[SE] g/cm^2^ = 0.009[0.0002]; Figure 4A).

Prevalence of the parasites *P. marinus* and *H. nelsoni* was low, precluding formal analyses. *P. marinus* infections were detected only in four cohorts (11 oysters) at the GA site (Figure S1A), and *H. nelsoni* infections were detected only in one cohort (1 oyster) in GA, and in two cohorts (4 oysters) in NC (*N* = 115–117 oysters per site; Figure 4B).

### 3.2 Evidence for local adaptation in survival

#### 3.2.1 Home vs. Away (HA) and Local vs. Foreign (LF)

**Figure 5:**
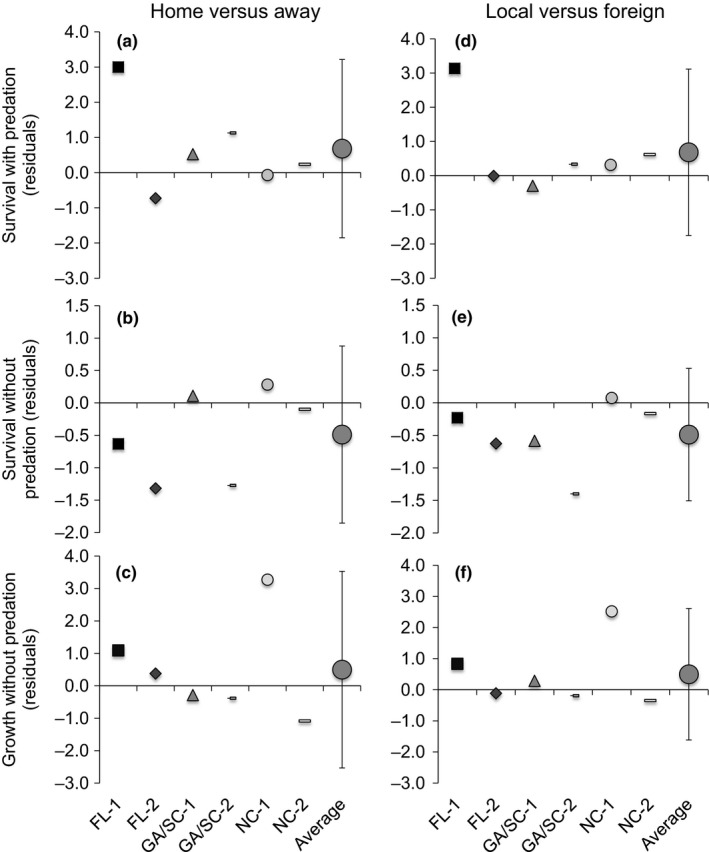
Home vs. Away (A‐C) and Local vs. Foreign (D‐F) metrics for oyster survival in the (A,D) partial cage and (B,E) cage treatments and (C,F) oyster growth in the cage treatments. Symbols indicate oyster cohort: Squares = FL‐1; Diamonds = FL‐2; Triangles = GA/SC‐1; Small dash = GA/SC‐2; Circles = NC‐1; Long dash = NC‐2. The residuals of survival after accounting for initial oyster size and the residuals of growth after accounting for initial oyster size and the number of surviving oysters are presented. The mean (+95% CI) across cohorts is presented in the large, dark gray circles

In the presence of predation, the FL‐1 cohort had higher survival at home than away, but the HA contrast was not significant overall (Figure 5A). Similarly, the LF contrast was not significant, with only the FL‐1 cohort having a local vs. foreign advantage (Figure 5D). The HA test in the absence of predation showed a much different pattern, with FL cohorts having lower survival at home and NC cohorts having slightly higher survival at home, but the effect across all cohorts was not significant (Figure 5B). Similarly, the GA cohorts had lower survival in GA compared to foreign cohorts in the absence of predation, but there was no LF difference overall (Figure 5E). There was no consistent HA or LF effect on oyster growth in the absence of predation (Figure 5C,F); for example, the NC‐1 cohort exhibited a home growth advantage and a local growth advantage, but the NC‐2 cohort showed the opposite response.

#### 3.2.2 Sympatry vs. Allopatry (SA)

The SA effect for survival in the absence of predation tended in the opposite direction of local adaptation (SA *F*_1,9_ = 4.43, *P* = .06): Cohorts had slightly higher survival in allopatry than sympatry. The SA effect was not significant for survival in the presence of predation or growth in the absence of predation.

#### 3.2.3 Distance

**Figure 6:**
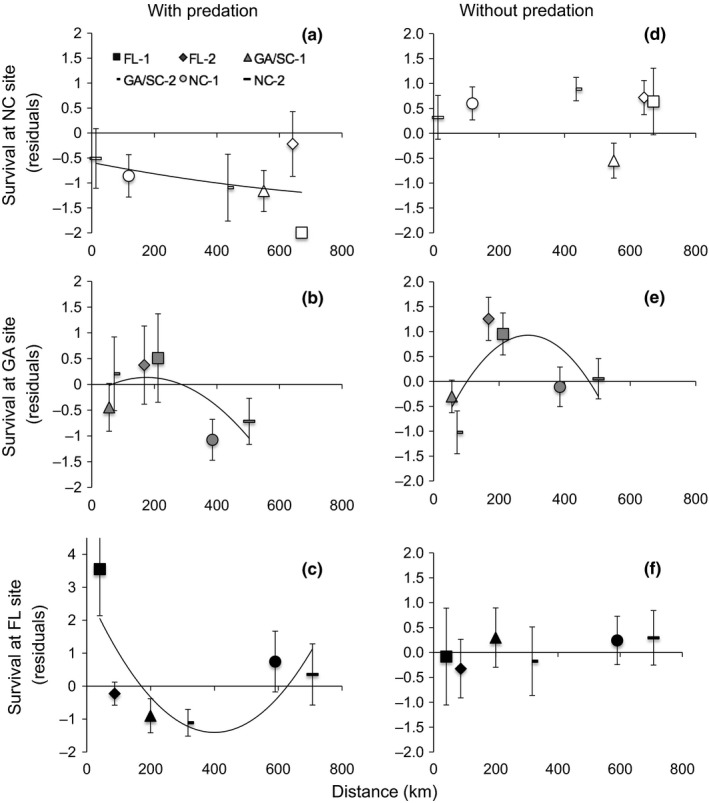
Mean (±SE) oyster survival, presented as the residuals after accounting for initial size differences, versus distance of the broodstock collection site to the experimental site in the (A‐C) partial cage and (D‐F) cage treatments. Open symbols (A,D) indicate the NC experimental site; gray symbols (B,E) indicate the GA experimental site; black symbols (C,F) indicate the FL experimental site. Symbols indicate oyster cohort: Squares = FL‐1; Diamonds = FL‐2; Triangles = GA/SC‐1; Small dash = GA/SC‐2; Circles = NC‐1; Long dash = NC‐2

Distance was a significant predictor of cohort survival (but not growth) at each experimental site, though the shape and direction of these relationships varied. Survival in the presence of predation had a unimodal relationship, with lowest survival in cohorts from intermediate distances at the NC (quadratic model chi‐square *P* = .04, *R* ^2^ = .16; Figure 6A) and FL (quadratic model chi‐square *P* < .0001, *R* ^2^ = .54; Figure 6C) sites, yet highest survival in cohorts from intermediate distances at the GA site (quadratic model chi‐square *P* = .01, *R* ^2^ = .43; Figure 6B). In contrast, survival in the absence of predation had no relationship with distance from collection to experimental site at the NC (chi‐square *P* > .33; Figure 6D) or FL (chi‐square *P* > .25; Figure 6F) sites, and peaked at intermediate distance at the GA site (quadratic model chi‐square *P* < .0001, *R* ^2^ = .48, *P* < .001; Figure 6E).

### 3.3 Evidence for countergradient variation in growth rate

Analyzing experimental sites separately, home latitude was not a significant predictor of growth at any site (linear or quadratic chi‐square *P* > .32).

## 4 Discussion

We conducted one of the few tests of local adaptation in a marine system using a field reciprocal transplant experiment with laboratory‐reared individuals from six different populations. Despite previous results showing little population genetic structure of oysters within the SAB using neutral markers (Diaz‐Ferguson et al., 2010), we found substantial GxE effects on juvenile oyster survival in both the presence and absence of predation, and on growth in the absence of predation. However, there was little evidence that cohorts were adapted to their home experimental site; in fact, the sympatric–allopatric (SA) comparison for survival in the absence of predation was more consistent with maladaptation (i.e., higher survival in allopatry than sympatry). Analyzing the effects of distance of the broodstock origin from the experimental sites confirmed the lack of local adaptation: Cohorts from sites closest to the experimental site did not consistently have higher survival or growth than those from farther away.

Despite differences in growth across cohorts and sites in the absence of predation, we did not find evidence for countergradient selection (Sanford & Kelly, 2011). Cohort home latitude was not a significant predictor of growth at any experimental site. These results are in stark contrast to the many examples of faster growth in marine populations from higher latitudes (Sanford & Kelly, 2011), particularly at warmer temperatures (Conover & Present, 1990), such as in summer when our experiment was conducted. It is possible we would have seen different growth responses during times when temperatures are more variable across sites (e.g., fall and winter). Alternatively, growth differences may take longer to emerge. Consistent with both of these explanations, a prior study of oyster populations encompassing multiple seasons in the mid‐Atlantic found growth patterns consistent with countergradient variation (Dittman, Ford, & Haskin, 1998). Our data suggest that countergradient variation is not an important factor influencing juvenile oyster growth rates within the SAB in the summer, but we cannot rule out its influence over longer time periods or at other times of year.

In addition to G*E effects on juvenile oyster survival and growth, we also found strong differences among cohorts (G effects) in condition in the absence of predation. These results are consistent with prior findings of substantial trait and performance variation across oyster populations, particularly in hatchery‐bred populations (Dittman et al., 1998; Pernet et al. 2008, Proestou et al., 2016). For instance, multiple lines of oysters have been developed for disease resistance in hatcheries in response to disease‐induced losses in aquaculture and restoration efforts (Ford & Tripp, 1996), and some of these lines performed consistently well across transplant sites in New England (Proestou et al., 2016).

The lack of local adaptation in our study differs from the few other tests of local adaptation in oysters (Burford et al., 2014; Eierman & Hare, 2013; Bible & Sanford, 2016). Each of these prior efforts targeted populations that differed genetically (Burford et al., 2014) or that occurred in distinct environmental conditions (along a salinity gradient; Eierman & Hare, 2013; Bible & Sanford, 2016). In contrast, we tested populations within a region with little population genetic structure which were selected to ensure similarity in characteristics such as salinity and tidal inundation (Byers et al., 2015), and the lack of extreme populations may have contributed to the absence of local adaptation (Bible & Sanford, 2016; Rice & Mack, 1991). Our study also encompassed a greater geographic range than previous efforts; although this larger spatial scale may have limited our ability to detect very fine‐scale differentiation (e.g., Hays, 2007), it is consistent with the scale at which adaptation commonly occurs (Sanford & Kelly, 2011). Further, oyster reef restoration efforts are being conducted throughout this range and elsewhere in the coastal USA. For areas experiencing recruitment failure, comparing the performance of local vs. more distant broodstock could assist future oyster reef restoration efforts.

Another unique feature of our experimental design was the ability to disentangle the effects of predation vs. other sources of mortality. Despite the high predation rates across our experimental sites, we did detect significant G*E interactions in the presence of predation, suggesting that variation in shell shape and/or size across cohorts may alter vulnerability to predation at some sites. For instance, we found evidence at one site in FL that the FL‐1 cohort may be adapted to avoid predators (i.e., the HA and LF contrasts for the FL‐1 cohort were positive in the presence of predation). We also detected G*E interactions in the absence of predation, yet the relative performance of cohorts at a given site differed from when in the presence of predation. Thus, predation and environmental characteristics that induce mortality both appear to interact with cohort identity to influence juvenile oyster survival. Unfortunately, the high predation rates in the no cage and partial cage treatments prevented us from comparing potential G*E in growth or condition in the presence and absence of predation. However, adaptation to the biotic environment may be common and deserves greater attention (Rua et al., 2016; Thompson, 1999).

When protected from predation (as in oyster aquaculture efforts, refugia created by the interstitial spaces of oysters, and/or naturally low predation sites; e.g., Garland & Kimbro, 2015), the potential for local adaptation in survival is higher if environmental conditions vary consistently and significantly across sites (c.f., Dittman, 1997; Dittman et al., 1998). Although we cannot rule out other environmental variables, our sites show minimal variation in salinity and temperature in the summer (Byers et al., 2015), and variation in tolerances resulting from differential selection would not necessarily be expected to manifest during the season when this experiment was conducted. Parasite prevalence also did not appear to explain variation in caged oyster survival across cohorts and sites: Our results suggest that oysters had more *H. nelsoni* exposure at the NC site and more *P. marinus* exposure at the GA site, yet survival was not consistently lower at these sites. The duration or timing of our experiment may have reduced the likelihood that our experimental oysters contracted parasite infections or that the parasites reached patency in their hosts: Disease‐related mortality and spread are highest in September and October, after our experiment ended. However, infection in juveniles can occur within days, and parasites are present in the water in summer when our experiment occurred (Burreson & Ford, 2004; McCollough, Albright, Abbe, Barker, & Dungan, 2007), suggesting that our parasite results are not merely an artifact of experimental design. It remains unclear what specific factors contributed to the differences in survival across cohorts and experimental sites in our cage treatments.

Consistent with many reciprocal transplant studies testing multiple populations (Leimu & Fischer, 2009; Sanford & Kelly, 2011), we did not find evidence for consistent local adaptation across all populations. Because we did not transplant cohorts into the exact sites where the adult broodstock were collected, it is possible that local adaptation occurs at a finer scale than tested here (i.e., less than 10‐30 km; Marshall et al., 2010; Richardson et al., 2014). Salmonid populations can exhibit local adaptation at scales of a few km (Taylor, 1991), and algae can be locally adapted along an intertidal gradient (Hays, 2007). Yet the pelagic larval phase of the eastern oyster spans approximately 2 weeks, which likely results in broader dispersal rates and reduces the probability that local adaptation is occurring in this species at fine spatial scales. In addition, although we used first‐generation individuals produced in a common hatchery environment for our reciprocal transplant experiment, persistent maternal effects resulting from parents collected under different environmental conditions may still have influenced our results (Sanford & Kelly, 2011). Future experiments that raise individuals through two or more generations in the laboratory are needed to rule out such maternal effects (Kawecki & Ebert, 2004; Sanford & Kelly, 2011).

Oysters are the focus of considerable restoration efforts (Grabowski et al., 2012), and common restoration practices include the outplanting of juvenile oysters (Bayraktarov et al., 2016). In addition, oysters are commonly produced from broodstock in commercial hatcheries and outplanted widely for aquaculture. Information regarding adaptive genetic variation and the degree of local adaptation is critical for guiding selection of source populations for restoration and aquaculture efforts (Marshall et al., 2010; Sanford & Kelly, 2011). Our results clearly demonstrate that oyster populations in the SAB are not a homogenous stock in terms of juvenile survival, growth, or condition, and importantly, cohorts from local broodstock do not consistently have higher fitness than those from farther away. Although some cohorts did have higher condition than others, such information is rarely available for natural populations prior to restoration. Further, the significant GxE effects on oyster survival and growth preclude using fitness in a particular environment as a reliable indicator of fitness at other sites (Conover & Schultz, 1995). Because of this lack of predictability and recent results demonstrating increased recruitment with increasing juvenile cohort diversity (Hanley et al., 2016), oyster restoration and aquaculture practices may benefit from incorporating multiple oyster cohorts into their efforts.

Understanding interactions of genes and the environment is key to population and community dynamics. Transplant experiments of multiple populations across sites along natural environmental gradients offer a powerful tool for examining the potential for local adaptation to mitigate the effects of climate change in natural populations (Anderson, 2016). For instance, forestry provenance trials demonstrate widespread local adaptation of tree populations along climatic gradients even with high levels of gene flow (Savolainen, Pyhajarvi, & Knurr, 2007). However, comparable experiments are underutilized for widely distributed species in marine systems (Sanford & Kelly, 2011), despite evidence for local adaptation in the ocean (Sanford & Kelly, 2011; Sotka, 2005). Furthermore, few field tests of G*E interactions in terrestrial or marine systems focus on early life history stages (but see Trussell 2000, Anderson et al., 2015), which are typically under the strongest selection pressure (Flatt & Heyland, 2011), especially at a regional scale with multiple populations. Addressing gaps in our knowledge of adaptive genetic variation in natural populations informs population responses to environmental change, including how uniform these responses may be across space. This understanding takes on special urgency in the face of changing climate (Anderson, 2016).

## Data accessibility

Data available from the Dryad Digital Repository: http://dx.doi.org/10.5061/dryad.8pm7h.
