# Supplementary material for: Genetic by environmental variation but no local adaptation in oysters (Crassostrea virginica)
Source: Ecol Evol. 2016 Dec 22;7(2):697–709. doi: 10.1002/ece3.2614 (PMC5243187; doi:10.1002/ece3.2614)

Genetic by environmental variation but no local adaptation in oysters (*Crassostrea virginica*)

A. Randall Hughes^1^*, Torrance C. Hanley^1^, James E. Byers^2^, Jonathan H. Grabowski^1^, Jennafer C. Malek^2^, Michael F. Piehler^3^, David L. Kimbro^1^

^1^Marine Science Center, Northeastern University, Nahant, MA 01908; ^2^Odum School of Ecology, University of Georgia, Athens, GA 30602; ^3^Institute of Marine Sciences, University of North Carolina at Chapel Hill, Morehead City, NC 28557; *rhughes@northeastern.edu

This information is in support of an article published in Ecology and Evolution.

Supplemental Figure 1. *P. marinus* prevalence was only detected at the GA experimental site and did not vary across oyster cohorts.


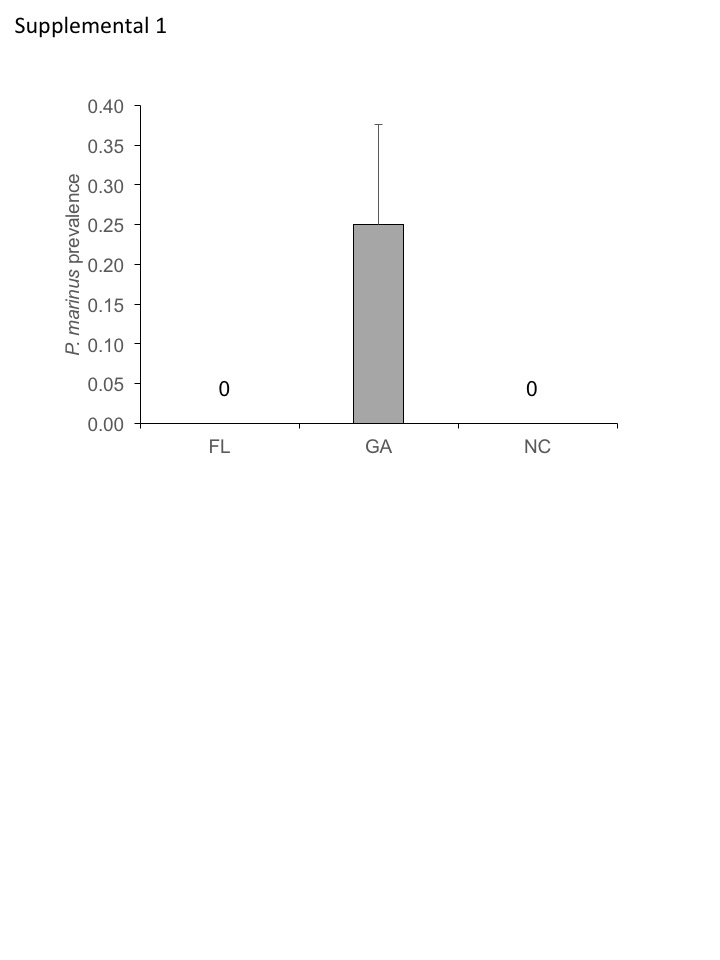

Supplement: Supplementary file 1 [file ECE3-7-697-s001.docx]
